# Effect of White Cabbage Intercropping with Aromatic Plant on Yield, Mineral and Biochemical Composition

**DOI:** 10.3390/plants13131870

**Published:** 2024-07-06

**Authors:** Armina Morkeliūnė, Neringa Rasiukevičiūtė, Lina Dėnė, Edita Dambrauskienė, Laisvūnė Duchovskienė, Alma Valiuškaitė

**Affiliations:** Lithuanian Research Centre for Agriculture and Forestry, Institute of Horticulture Babtai, 58344 Kėdainiai, Lithuania; neringa.rasiukeviciute@lammc.lt (N.R.); lina.dene@lammc.lt (L.D.); alma.valiuskaite@lammc.lt (A.V.)

**Keywords:** intercropping, cabbage macronutrients, sustainable horticulture, vegetable quality

## Abstract

The growing demand for higher-quality food production in smaller soil areas points to optimized land use. Intercropping has the potential to increase yield, reduce pests and diseases, and boost biodiversity. This study, conducted at the Institute of Horticulture, Lithuanian Research Centre for Agriculture and Forestry, from 2017 to 2019, aimed to determine the effect of white cabbage intercropping with aromatic plants, calendula, French marigold, thyme, and sage on yield, mineral and biochemical composition. Aromatic plants are known to reduce the occurrence of pests and diseases, so this study aimed to determine whether aromatic plants affect the yield, mineral, and biochemical composition of white cabbage. The two-year observations demonstrated that aromatic plants did not affect or slightly affect the mineral composition of cabbage’s primary macronutrients (N, P, K, Mg, and Ca). Cabbage’s dry matter, sugars, and ascorbic acid content vary when grown intercropped with aromatic plants compared to monoculture. Although the results were comparable, sugar concentration was lower in all cabbage combinations than in monoculture. Lower nitrate levels were detected in cabbage monoculture, probably due to agro-meteorological circumstances. The highest cabbage yield was achieved by intercropping with thyme (7.25 t/ha) compared to monoculture (6.81 t/ha) in 2018. It was found that intercropping with aromatic plants had little effect on the biochemical composition of white cabbage. The study results suggest that French marigold and thyme can be grown together with white cabbage to improve the phytosanitary of vegetables without compromising the biochemical quality of the cabbages. However, the influence on biochemical composition, especially on the nitrate and glucosinolate levels, should be examined further, providing valuable insights for future research in this field.

## 1. Introduction

Environmental and climate-friendly farming methods in agriculture are promoted by European Union directives (European Green Deal), which encourage greater implementation of ecological measures. Intercropping is valued for its potential to increase yield quality, reduce pests and diseases, benefit the soil (legumes), boost biodiversity, and optimize land use. However, intercropping systems are traditional farming practices. Studies have shown this tool’s effectiveness, but it is not commonly employed in agriculture [1,2,3,4,5,6,7,8]. Insights have revived the intercropping method [9]. Given the growing population and food demand, growing larger yields or higher-quality food production in a smaller soil area is essential [10,11]. Furthermore, intercropped systems must be profitable and economically viable. The crops share the same productive resources, space and time, which leads to efficient resource use [9].

Additionally, intercropping has the potential to be used as an alternative pest control technology, especially nowadays, as there is a shift towards sustainable plant protection measures and a reduction in the use of chemical pesticides. Pesticide residues in fruits, vegetables, and other agricultural products harm humans, as persistent environmental pollution damages ecosystems, microorganisms, and animals. Intercropping is a promising ecological intensification model for increasing productivity while reducing environmental impact [8,11].

A thorough understanding of the subject (crops grown, their compatibility, physiological processes, etc.) is required to capitalize on the benefits of adopting the intercropping system for agroecological processes and generate positive intercropping results [6]. The literature indicates the economic potential of maize–cabbage intercropping by selecting the optimal plant combinations [12]. Leguminous crops may be used as an organic means of fertilizing cabbage [13]. Crop diversification increases nutrient availability by affecting soil pH, aggregate stability, and microbial community [14]. Many literature sources can be found that mention the benefits of intercropping on cabbage. An abundance of different crops growing in the same field can improve target quality parameters like cabbage head size and maintain yield per unit area [15]. Vegetables such as *Brassica oleracea* are commercially useful, widely farmed, and fundamental components of the human diet [16,17]. Vitamins, organic acids, glucosinolates, anthocyanins, amino acids, minerals, chemopreventive, anti-cancerogenic, and other bioactive components can all be found in cabbages [18,19,20]. Cabbage biochemical composition depends on cultivation practices, the appearance of weeds [21], genotype and environmental eco-physiological factors, growth, transportation, and storage conditions [20,22]. The impact of various intercropping arrangements on the nutritional contents of cabbage was investigated by Guvenc et al. in 2006 [4]. The amount of nitrogen, phosphorus, potassium, calcium, magnesium, and iron in cabbage was not considerably impacted by cropping patterns. Plant resistance to diseases such as *Phytophthora brassicae*, *Plectosphaerella cucumerina*, and *Botrytis cinerea* is influenced by glucosinolates [23]. It is essential to ensure high-quality growing conditions for cabbages to increase the added value of these vegetables. Furthermore, cabbage’s composition has many health-beneficiary compounds, and providing them at the highest possible rates would improve public health [22]. Cabbage is known to scavenge nitrogen from the soil and accumulate nitrates in its tissues, an undesired feature in the final product, so it is important to limit their absorption [13]. Literature sources [2,24,25,26,27,28,29] have linked intercropping to pest control, and some [3,30,31] have investigated aromatic plants. Numerous metabolites possessing antibacterial, antioxidant, insect-repellent, and herbicidal qualities are accumulated by aromatic plants [32]. Aromatic plants are extensively spread plant species that occur naturally; most of these plants are cultivated for their essential oils. Aromatic plants have the potential to enhance crop yield by maintaining soil biological activity, minimizing tillage, and promoting biodiversity within the ecosystem. Moreover, aromatic plants help decrease the occurrence of pests and diseases [33].

Intercropping tropical basil with cabbage resulted in a decrease in the population of insect pests [2], whereas intercropping fenugreek with faba beans led to a reduction in weed infestation [30]. Consequently, intercropping with aromatic plants might result in the development of pesticide characteristics, leading to a higher yield by reducing production losses. Intercropping has the potential to inhibit pest infestations and decrease reliance on chemical pesticides.

A key component of this agricultural approach is the influence on the quality of vegetables in accordance with the European Union’s guidelines for implementing sustainable farming initiatives. The Common Agricultural Policy (CAP) integrates social, economic, and environmental policy matters, scientific research, and creative strategies to establish a sustainable agricultural system inside the European Union (EU) [11].

There is still a need for a comprehensive analysis of the impact of intercropping on the biochemical quality of vegetables. Additional data are required regarding the alterations in nutritional value, mineral composition, and other qualitative aspects of cabbages cultivated by intercropping methods. After assessing this effect, it is possible to predict the advantages and disadvantages of intercropping with different plant species, determine when it is worth applying this method, or decide which plants to choose. Intercropping and mixed crops affect yield quality, enhance soil fertility [5], and can improve soil quality [5,34], increasing the biodiversity in the agroecosystem [26]. A literature review revealed the importance of simultaneously studying the mineral and nutritional composition of cabbage, as plants cultivated in the same soil and field have an impact on each other.

It is advised that processing pesticidal volatiles and intercropped aromatic plants has a neutral or favorable effect on crops when applied in agricultural techniques. As flowers, French marigold (*Tagetes patula* L.) is widely cultivated. In addition, it serves as a beverage, repellant, antiseptic, and medication. French marigold contains antifungal properties in agriculture that help to prevent early blight of tomatoes. Although thyme (*Thymus vulgaris* L.) and sage (*Salvia officinalis* L.) essential oils are plant-derived products with many uses in the food, perfume, and medical industries, recent studies on essential oils have shown that they may also have antibacterial properties. Intercropping with calendula and French marigold significantly reduced cabbage pests and diseases, and aromatic plants are a source of nectar and pollen. Aromatic plants were chosen for their well-known antifungal activity against plant pathogens, as shown in our previous studies [35,36,37,38,39].

This research aimed to determine the effect of white cabbage intercropping with aromatic plants, calendula (*Calendula officinalis* L.), French marigold (*Tagetes patula* L.), thyme (*Thymus vulgaris* L.), and sage (*Salvia officinalis* L.), to evaluate its impact on yield, mineral and biochemical composition.

## 2. Results

Several cabbage intercropping combinations were evaluated for yield, mineral composition, dry matter, ascorbic acid, sugar content, nitrate amount, and glucosinolates.

### 2.1. Cabbage Yield

**Figure 1:**
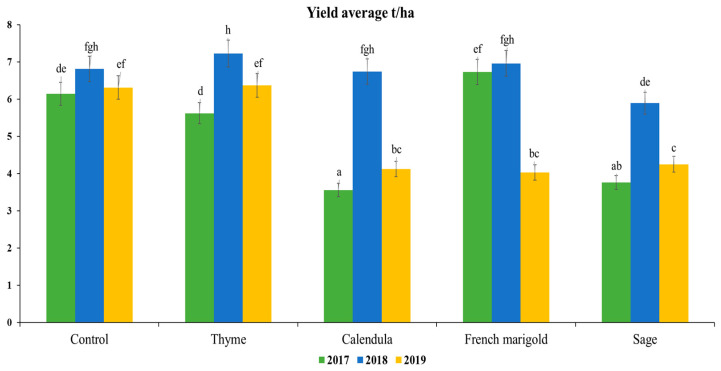
Yield average results in the experimental period 2017–2019. The same letter indicates no significant differences between yield according to Tukey’s HSD test (*p* < 0.05).

The yield of cabbage intercropped with aromatic plants is presented in Figure 1. Data revealed that during the study, the yield of the monoculture of cabbage (control CM) did not change significantly and remained stable during the research (6.14, 6.81, and 6.31 t/ha). In 2017 and 2019, the CTH yield was the same as the control (Figure 1). In 2018, CTH produced the maximum yield (7.25 t/ha). Compared to other treatments, CCL and CSG in 2017 significantly decreased cabbage yield (3.56 and 3.76 t/ha). Cabbage yield intercropped with CFM increased by 6.73 t/ha in 2017. However, the productivity of cabbage was reduced in 2019, and the results were similar to those of CCL and CSG. The yield average of these three treatments varied from 4.03 to 4.25 t/ha in 2019. Cabbage yield was similar and varied from 6.74 to 7.22 t/ha in all treatments, whereas the yield of CSG was 5.89 t/ha in 2018. According to the three-year data, CTH did not significantly change the cabbage yield.

### 2.2. Mineral Composition

Analysis of the mineral composition was carried out in 2017–2018. Intercropping with aromatic plants resulted that obtained differences are negligible (Table 1). CSG and CTH had the highest quantity of selected minerals in 2017. The nitrogen and calcium percentage values were higher in CTH compared to other treatments in 2017. A slightly higher percentage of nitrogen and phosphorus was detected in CM (0.18 and 0.020%, respectively) compared to all other treatments in 2018. CTH accumulates more potassium and calcium than other treatments in our research (respectively, 0.18% and 0.041%) in 2018. The magnesium percentage was recorded from 0.0083% in CSG to 0.0096% in CFM. Intercropping results varied between the treatments and were close to CM in both years.

### 2.3. Dry Matter and Ascorbic Acid

Dry matter and ascorbic acid values in cabbages were analyzed in 2017–2018 (Table 2). Similar dry-matter percentages were observed in all treatments, but the results slightly varied between years. The highest dry-matter percentage was found in CTH and the lowest in CCL (respectively, 7.00 and 5.49%) in 2017. Meanwhile, the percentage of dry matter was 6.16% in CM. The percentage of dry matter in 2018 varied from 5.97% in CFM to 6.96% in CM. The ascorbic acid levels in 2017 in CTH was significantly higher, 5.00 mg/100 g, whereas in CCL, it was 2.60 mg/100 g. Overall, a higher ascorbic acid content was determined in 2018 than in 2017. The ascorbic acid 2018 in CSG was lowest (4.73 mg/100 g), whereas in CM, it was highest (5.81 mg/100 g). Intercropping CCL and CM resulted in a higher amount of ascorbic acid compared to all other treatments in 2018.

### 2.4. Sugar Content and Nitrates Quantity

Sugar values were observed in 2017–2019 and are presented in Table 3. Significant differences were observed. In 2017, a much larger amount of sugars was found in CM (4.35%) and CFM (4.17%), followed by CCL, which accumulated the least amount of sugar, 2.52%, in 2017. Nevertheless, the results were close to those obtained in CM treatment in the subsequent years. Sugar content was higher in CM in all three years.

The most significant differences in results were obtained from nitrate analysis (Table 3). It was observed that CSG (1564 mg/kg), CTH (1628 mg/kg), and CCL (2139 mg/kg) had higher nitrate levels in 2017, whereas different results were observed in these treatments in the following year. CFM had the lowest content of nitrates (722 mg/kg), which was significant. The highest content of nitrates was found in CTH (1606 mg/kg) in 2018. However, the nitrate content was considerably lower in all treatments in 2019 than in 2018. Overall, intercropping CSG, CTH and CFM resulted in higher nitrate levels than in CM.

### 2.5. Glucosinolates

Glucosinolate values were observed in 2017–2019 and are presented in Table 4. CTH accumulated more glucosinolates than other treatments in three years (1.24, 1.08, and 1.16%, respectively). The most significant differences in results were obtained from CCL (1.39%) in 2018 and CSG treatment (1.39%) in 2019. However, these results differed significantly from the data obtained the following year.

## 3. Discussion

We found that qualitative research on the composition of crops following intercropping is necessary. This study examined the effects of intercropping French marigold, thyme, sage, and calendula on various parameters such as yield, dry matter, ascorbic acid, sulfur content, nitrate amount, and glucosinolates. Our findings concurred with those of Guvenc et al. [4], who found no significant differences in the nitrogen, phosphorus, potassium, calcium, and magnesium levels of cabbages intercropped with cos and leaf lettuce, onion, radish, and beans between experimental years. Ulbrich et al. [32] investigated cabbage and white cabbage intercropping systems with *Mentha* plants and found *Mentha* plants helpful for *Brassica* vegetation, triggering the leaf development of cabbage even cultivated under defined conditions (water content, soil, nutrients). They also investigated and evaluated minerals in Chinese cabbage and compared growing technologies. Pokluda [21] observed mineral content differences between cultivars (except magnesium) and determined positive correlations between dry matter–potassium content and dry matter–nitrates. Kim et al. [10] verified that the mineral composition of Chinese cabbages differed depending on whether they were cultivated using organic, natural, or conventional agricultural methods. In our study, CM accumulated the largest amount of sugars in all experiments. In 2018, all treatments had decreasing sugar content. We assume this might be related to weather factors like reduced precipitation and an average temperature increase. In contrast, the 2018 results show that the total sugar levels in Chinese cabbages grown using conventional and organic agricultural methods are comparable [10]. Contrary to the sugar concentration, ascorbic acid values were higher in 2018 than in 2017. Our observed dry matter, ascorbic acid, and sugars were sufficiently lower than cabbages fertilized with biofertilizers and organic fertilizers [24]. Stein et al. [13] state that *Leguminous* crops may serve as additional organic (natural) fertilizers to reduce weeds, provide additional nitrogen to the soil, reduce nitrate leaching, and improve soil properties. Thus, intercropping with aromatic plants requires fertilization for higher-quality vegetables.

CCL and CSG reduced cabbage yield. Larger plants (calendula, sage) stopped the rapid growth of cabbage at the beginning of vegetation. According to the three-year data, thyme did not change the cabbage yield. However, Jankowska et al. [3,40] observed a positive effect of French marigolds and calendula on the cabbage and carrot yield. Juventia et al. [26] observed that wheat and *Brassica* intercropping systems were more productive when grown in alternating strips. Intercropping with marigolds increased tomato fruit yield [14].

The study conducted by Debra et al. [17] shows that intercropping cabbages with onion and garlic has a beneficial impact on reducing insect prevalence and increasing cabbage output, as compared to cabbage monoculture. Carrillo-Reche et al. [6] conducted a comprehensive investigation of 76 studies, indicating that intercropping systems reduced cabbage productivity by 7% and pest injury by 48% relative to sole cabbage systems. In our research, the three-year yield average in CM was higher and more stable than in other treatments. Lepse et al. [7] assert that the presence of French marigold had a profound negative impact on the cabbage plants in their study, making it difficult to accurately evaluate the true effect on cabbage plant growth. Our investigation demonstrates that the presence of French marigold did not inhibit the growth of nearby plants and conditions suitable for developing cabbage.

In 2017, we observed that at the beginning of vegetation, CSG and CCL inhibited cabbage growth. We found more nitrates in 2017, and the high nitrate content in vegetables is an undesirable indicator. Additionally, CCLs in 2018 were of higher quality than in 2017. Significantly higher amounts of nitrates were determined in our study compared to cabbages fertilized with biofertilizers and organic fertilizers [24]. The obtained values were closer to those in Pokluda’s [21] study. In contrast to 2017 and 2018, the nitrate content in our treatments (CTH, CSG, CCL, and CM) was much lower in 2019. Different meteorological conditions could account for variations in nitrate levels; for example, June 2018–2019 saw a dryer month with above-average temperatures. Under conditions of sufficient lightning, vegetables have a tendency to collect lower levels of nitrates. Due to less sunlight in 2017 compared to 2018 and 2019, cabbages exhibited heightened sensitivity to the shade cast by tall and rapidly growing calendulas. This led to an elevation in nitrate levels.

The research on glucosinolates indicates their potential role in the defense of *Brassica napus* against *Alternaria brassicae*. Glucosinolates are employed by plants as a means of defense through the signaling of jasmonic acid, salicylic acid, and ethylene hormones [14]. Wermter et al. [20] claim that lack of precipitation and higher temperatures lead to higher levels of glucosinolates. Francisco et al. [16] confirmed that a low average precipitation and a high average temperature during the vegetation period increase the number of glucosinolates and vitamin C in cabbage. Our observed glucosinolate values significantly differed between experimental years and all treatments. Thus, it was not easy to assess the influence of meteorological conditions.

Overall, the findings demonstrated no impact of aromatic plants on the biochemical composition of white cabbage. Still, further investigation is required to fully understand the impact of French marigold and thyme on nitrate and glucosinolate concentrations. Intercropping aromatic plants may enhance the biodiversity of the agroecosystem when compared to CM. In addition, the growing demand for environmentally sustainable development encourages the rapid implementation of technical modifications. Applying intercropping techniques can lead to the production of better output and the promotion of sustainable land management.

## 4. Materials and Methods

### 4.1. Field Trial

The investigation was conducted in the experimental fields (55.081052, 23.806630) of the Institute of Horticulture, Lithuanian Research Centre for Agriculture and Forestry (LAMMC) from 2017 to 2019. The soil of the experimental site was Epicalcari-Epihypogleyic Cambisol, CMg-p-w-cap, with a clay loam texture. The arable layer was weakly alkaline (pH 7.1–7.3) with medium humus (1.95–2.67%). The agrochemical parameters of the soil before the experiment are shown in Table 5.

**Figure 2:**
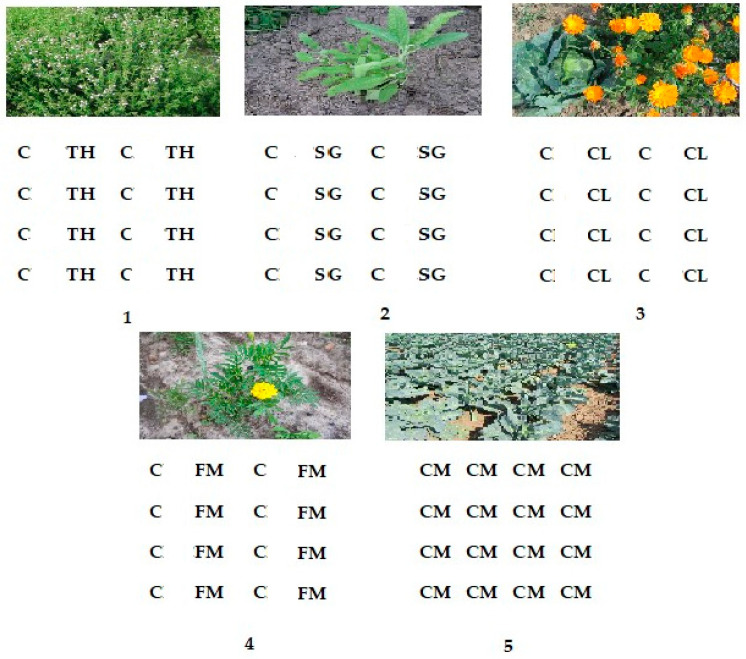
Schematic illustration of different treatments: (**1**) cabbage (C) with thyme (TH), (**2**) cabbage (C) with sage (SG), (**3**) cabbage (C) with calendula (CL), (**4**) cabbage (C) with French marigold (FM), and (**5**) cabbage monoculture (CM).

Figure 2 shows a schematic representation of the treatments. The intercropped plants were arranged in alternate rows (one row of cabbage alternating with one row of aromatic plant (70 × 40 cm)). Mechanical weed removal was used instead of chemical pesticide treatments. Fertilizers were not applied. The cabbages were harvested when they reached marketable size and quality at the end of September.

### 4.2. Plant Material

Aromatic plants such as calendula, French marigold, thyme, and sage were interplanted with the main crop, white cabbage (*Brassica oleracea* var. *capitata*) ‘Krautman F1’ plants. The compared treatments are: (1) cabbages intercropped with thyme—CTH; (2) cabbage with sage—one row of cabbage and one row of sage—CSG; (3) cabbage with calendula—CCL; (4) cabbage with French marigold—CFM; and (5) cabbage monoculture—control—CM. Our previous studies helped us select aromatics for this study [32,33,34,35,36]. Their effects on strawberries, white cabbage, carrots, and other horticultural plants have previously been recognized to have insecticidal and antifungal qualities.

The study selected the ‘Krautman F1’ type of white cabbage because of its popularity and extensive cultivation. These cabbages are abundant and suitable for fresh vegetable markets, and the harvest of the second rotation is ideally stored for about four months.

Calendula (*Calendula officinalis* L. annual, belongs to *Asteraceae*, height of plant 50–60 cm) was sown directly into the field at the end of 24–28 April (sowing rate 8 kg/ha), French marigold (*Tagetes patula* L. belongs to *Asteraceae,* annual, height of plant 20–30 cm) (sowing rate 2 kg/ha) and cabbage were transplanted at the end of 27–30 May in 2017, 2018, 2019. Thyme (*Thymus vulgaris* L. belongs to *Lamiaceae*, perennial, height of plant 20–30 cm) and sage (*Salvia officinalis* L. belongs to *Lamiaceae*, perennial, height of plant 30–40 cm) were planted in 2017 (36,000 plants/ha). Thyme and sage plants were obtained from the Lithuanian Research Centre for Agriculture and Forestry (LAMMC) Institute of Horticulture (IH) collection fields. The experiment was arranged in a randomized design with 4 replicates.

### 4.3. Analysis

Cabbage yield and quality were observed during the experimental years. Yield evaluated: one cabbage weight and total yield per plot in t/ha. The 2017, 2018, 2019 cabbage yield was harvested at the end of September (BBCH 49).

**Figure 3:**
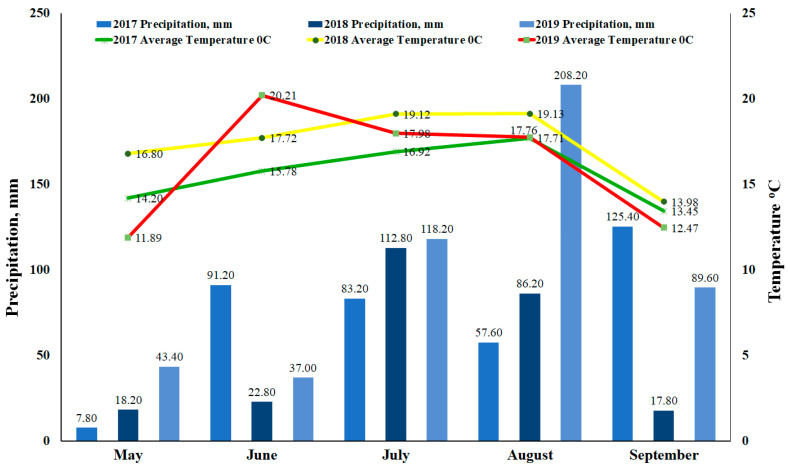
Monthly temperature and precipitation in the experimental area, May–September, 2017–2019.

Biochemical composition parameters were determined in the Agrochemical Research Laboratory, LAMMC. Dry matter (%) in cabbage was determined using methods set out in Commission Directive 71/393/EEB in 2017–2018. The samples were dried at 105 °C until constant weight. The total sugar content (%) was established based on the Luff Schoorl method—by directive 71/250/EEB from fresh material in 2017, 2018, 2019. Ascorbic acid (mg/100 g) was determined by Moore’s method by titration of 2,6-dichlorophenolindophenol sodium salt solution (Tilmans reagent) LST ISO 6557-2:2000 [41] in 2017–2018. Nitrates (NO_3_^−^, mg/kg) were analyzed using the ionometric method from fresh material in 2017, 2018, and 2019. Nitrate contents were calculated from fresh material based on No. 160/3–2841 methodological instructions for determining nitrates in crop production. The chemical composition of macronutrients in 2017–2018 in cabbage (dry material) were determined using following directives: nitrogen (N, %) was determined by the Kjeldahl method according to 72/199/EEB; potassium (K, %) was determined by the flame photometric method—71/250/EEB; phosphorus (P, %) was determined calorimetrically—71/393/EEB; magnesium (Mg, %) was determined by the atomic absorption spectrometric method—73/46/EEB; and calcium (Ca, %) was determined by the flame photometric method—71/250/EEB. The colorimetric method was used to determine glucosinolates in 2017, 2018, and 2019 from dry material according to ISO 9167:2019 standards [42]. Ten cabbages were collected from each treatment for biochemical analysis. The experiments where not irrigated; additionally, the meteorological conditions are presented in Figure 3.

### 4.4. Statistical Analysis

Before statistical processing, average values were analyzed based on the growth seasons. The data were analyzed using a one-way variance (*ANOVA*) procedure. Tukey’s HSD test estimated the significance of differences between treatments at *p <* 0.05. The data are represented as the means of the measurements.

### 4.5. Meteorological Data

Meteorological data were collected from weather station iMetos^®^ (Pessl Instruments, Malvern, VIC, Austria). The meteorological station is located 1.0 km from the trial field (Babtai, Kaunas distr.). Average temperature and total precipitation were recorded daily during the cabbage growing months (May to September) in 2017–2019 (Figure 3). Data collected from the weather station in 2017 showed that the temperatures in June, July and August were the lowest in all three years during the study. It should be mentioned that the amount of precipitation was lowest in May, July, and August. The temperatures in May–September 2018 were higher than in 2017, and the amount of precipitation was quite similar. Nevertheless, September 2018 was quite dry. Temperatures in 2019 were close to 2017, but the highest amount of precipitation was recorded in August, and it was twice as high as in both 2017 and 2018.

## 5. Conclusions

The mineral composition of cabbages, comprising calcium, magnesium, phosphorus, potassium, and nitrogen, remained unchanged or experienced minimal alterations due to the presence of aromatic plants. Intercropping cabbages with aromatic plants leads to notable variations in the dry-matter and ascorbic acid contents of the cabbages compared to CM. All cabbage combinations had lower sugar content than CM. However, the outcomes were not dissimilar from those of CM. In our investigation, the concentration of nitrates changed during the experimental years. The reduced nitrate levels were found in CM. CCL and CSG can reduce cabbage yield. Therefore, further research should verify the findings, especially for CCL and CSG. The influence of thyme and French marigold on nitrate and glucosinolate levels should be examined further. The studies’ findings suggest that thyme (*Thymus vulgaris* L.) and French marigold (*Tagetes patula* L.) could be intercropped with white cabbage to enhance phytosanitary conditions for vegetables without affecting the cabbages’ biochemical composition. We suggest conducting additional research using various species and cultivars to examine the impacts of intercropping.

## Figures and Tables

**Table 1:** Mineral composition of cabbage intercropped with aromatic plants.

Year	Treatment	Percentage (%)				
		N	P	K	Ca	Mg
2017	CM	0.1200 a	0.0160 c	0.1500 c	0.0360 b	0.0086 a
	CCL	0.1500 c	0.0150 b	0.1400 b	0.0360 b	0.0088 b
	CFM	0.1400 b	0.0170 d	0.1600 d	0.0350 a	0.0090 c
	CSG	0.1600 d	0.0180 e	0.1600 d	0.0360 b	0.0100 d
	CTH	0.1800 e	0.0170 d	0.1300 a	0.0410 c	0.0110 e
2018	CM	0.1800 b	0.0200 d	0.1700 c	0.0360 d	0.0091 d
	CCL	0.1600 a	0.0180 b	0.1600 b	0.0350 c	0.0089 c
	CFM	0.1600 a	0.0190 c	0.1700 c	0.0330 b	0.0096 e
	CSG	0.1600 a	0.0170 a	0.1500 a	0.0310 a	0.0083 a
	CTH	0.1600 a	0.0180 b	0.1800 d	0.0410 e	0.0088 b

Note. According to Tukey’s HSD test (*p* < 0.05); the same letter indicates no significant differences between intercropping.

**Table 2:** The content of dry matter and ascorbic acid of cabbage intercropped with aromatic plants.

Year	Treatment	Dry Matter (%)	Ascorbic Acid (mg/100 g)
2017	CM	6.16 b	2.80 b
	CCL	5.49 a	2.60 a
	CFM	6.91 d	2.80 b
	CSG	6.54 c	2.90 c
	CTH	7.00 e	5.00 d
2018	CM	6.96 e	5.81 e
	CCL	6.84 d	5.66 d
	CFM	5.97 a	4.76 b
	CSG	6.37 b	4.73 a
	CTH	6.77 d	5.63 c

Note. According to Tukey’s HSD test (*p* < 0.05); the same letter indicates no significant differences between intercropping.

**Table 3:** Sugar content and nitrates quantity of cabbage intercropped with aromatic plants.

Treatment	Sugar Content (%)			NO_3_^−^ (mg/kg)		
	2017	2018	2019	2017	2018	2019
CM	4.35 e	3.81 d	3.98 e	1006 b	1222 b	476 a
CCL	2.52 a	3.36 c	3.77 d	2139 e	1089 a	615 b
CFM	4.17 d	3.20 b	3.39 a	722 a	1333 c	1121 e
CSG	3.70 b	3.18 a	3.64 c	1564 c	1500 d	1042 d
CTH	3.91 c	3.35 c	3.45 b	1628 d	1606 e	983 c

Note. According to Tukey’s HSD test (*p* < 0.05); the same letter indicates no significant differences between intercropping.

**Table 4:** Glucosinolate quantity in cabbage intercropped with aromatic plants.

Treatment		Glucosinolates (%)	
	2017	2018	2019
CM	0.38 a	0.45 c	0.58 b
CCL	0.67 b	1.39 e	0.39 a
CFM	1.12 d	0.42 c	0.93 c
CSG	0.77 c	0.25 b	1.39 e
CTH	1.24 e	1.08 d	1.16 d

Note. According to Tukey’s HSD test (*p* < 0.05); the same letter indicates no significant differences between intercropping.

**Table 5:** Soil agrochemical parameters.

Year	pH	Organic Materials	Hummus	Organic C	N Total	P_2_O_5_	K_2_O	Ca	Mg
		%				mg/kg			
2017	7.3	3.91	2.67	1.55	0.13	200	150	14,908	4024
2018	7.2	3.60	2.38	1.38	0.12	308	181	4078	758
2019	7.1	3.22	1.95	1.13	0.12	271	212	10,340	2264

## Data Availability

The data presented in this study are available upon request from the corresponding author.
